# Combined mechanical ventilatory and mechanical circulatory support aids pulmonary vascular state in cardiogenic shock

**DOI:** 10.1186/s40635-025-00811-2

**Published:** 2025-10-15

**Authors:** Kimberly K. Lamberti, Elazer R. Edelman, Steven P. Keller

**Affiliations:** 1 Institute for Medical Engineering and Science, Massachusetts Institute of Technology, 77 Massachusetts Ave., Cambridge, MA 02139 USA; 2 Cardiovascular Medicine, Brigham and Women’s Hospital, Harvard Medical School, Boston, MA USA; 3 Pulmonary and Critical Care Medicine, Johns Hopkins University, Baltimore, MA USA

**Keywords:** Mechanical circulatory support, Mechanical ventilation, Right-left ventricular coupling, Positive end expiratory pressure

## Abstract

**Background:**

Percutaneous ventricular assist devices (pVADs) support patients in circulatory failure and increasingly concomitant respiratory failure. The presence of co-existent lung disease creates a management challenge due to cardiopulmonary interactions, especially when there is simultaneous mechanical ventilation and mechanical circulatory support. Enhanced understanding of the combined effects of these devices is necessary to better inform care for circulatory failure patients.

**Methods:**

A porcine model of titratable acute cardiogenic shock was used to quantify the effect of pVAD support on cardiac loading states in five intubated animals with positive pressure ventilation and varied intrathoracic pressure. Cardiovascular hemodynamics were assessed across positive end-expiratory pressure (PEEP) ramps in animals in health, health with pVAD, and pVAD-supported cardiogenic shock induced via coronary microembolization.

**Results:**

This study employed invasive physiological metrics and assessment of right and left ventricular press-volume loops to recreate classic Frank-Starling curves. Increased intrathoracic pressure altered transmural pressure in the ventricles and the pulmonary vasculature and resulted in decreased venous return and stroke volume while increasing end-diastolic pressure consistent with decreased ventricular compliance. In pVAD-supported cardiogenic shock, elevated PEEP enhanced left ventricular output and increased pulmonary vascular compliance in several animals, contrary to traditional decrements observed with elevated PEEP. The right ventricular functional response aligned with these varied responses in pulmonary vascular state.

**Conclusions:**

These results demonstrate that combined used of cardiopulmonary support devices in cardiogenic shock can create variable responses compared to classic physiological understanding. In pVAD-supported cardiogenic shock, an increase in ventilatory PEEP increased unloading from the heart and improved right ventricular function, counter to traditional findings. This demonstrates that combined use of these technologies could be leveraged to optimize a patient’s volume status in complex shock and provides promise for management of patients with cardiopulmonary failure requiring simultaneous use of mechanical circulatory support and mechanical ventilation.

**Supplementary Information:**

The online version contains supplementary material available at 10.1186/s40635-025-00811-2.

## Introduction

Percutaneous ventricular assist devices (pVADs) are increasingly relied on to augment perfusion for patients in cardiogenic shock [1–3]. Consisting of a catheter-mounted axial pump positioned across the aortic valve, the pVAD provides continuous blood flow from the left ventricle (LV) to the proximal aorta throughout the cardiac cycle [4]. Despite rapidly growing clinical adoption, there is incomplete understanding of the spectrum of physiological effects of pVAD use and factors that impact device operation [5]. Contributing to the challenge of optimal device use is the medical complexity of cardiogenic shock patients requiring mechanical circulatory support (MCS) [1, 6–9]. These patients frequently suffer from concomitant pulmonary disease which presents a unique management challenge due to interactions between the impaired heart and lungs that may affect the dynamics and efficacy of pVAD support [10–13].

The heart and lungs are intrinsically coupled through both their colocation within the thoracic cavity and their intertwined vascular connections [14]. Perturbations to the intrathoracic environment are transmitted to both organs and can affect their physiological state and function. Mechanical ventilation, the mainstay therapy for respiratory failure and most widely used support device in critically ill patients, can impact venous return and loading conditions of both ventricles through application of positive pressure to the lungs [15–17]. Introduction of the pVAD produces additional effects as it alters the loading states of both the LV and right ventricle (RV) due to series interactions through the pulmonary circulation and parallel interactions across the intervening septum [18, 19]. As pVAD and ventilatory support alter loading state on both ventricles, the effect of changes in intrathoracic pressure on the heart in the setting of pVAD support is critical to inform clinical management of these patients.

As the subject of study for decades, foundational concepts of heart–lung relationships were determined through evaluation of the healthy response using in vivo models and in small patient populations requiring ventilator support [20–25]. The emerging use of simultaneous mechanical ventilation and MCS in cardiogenic shock patients presents new questions in heart–lung interactions important for clinical care. The current study seeks to leverage advances in research technologies and improved assessment techniques to deepen understanding of the effects of dual use of mechanical ventilator and pVAD on heart–lung interactions. We employ a porcine model of cardiopulmonary intervention to assess the effect of pVAD support on the loading states of the RV and LV across changes in intrathoracic pressure. Further, we seek to expand appreciation of the effect of positive end-expiratory pressure (PEEP) variation on ventricular-ventricular coupling in the setting of left-sided MCS and to extend these findings to the clinical condition through induction of acute cardiogenic shock in the porcine model. Understanding the interactions of dual mechanical support with both pVAD and ventilator may provide new insights to improve clinical management of critically ill patients.

## Methods

### Animal preparation and data acquisition

Heart–lung interactions were assessed in a series of five acute animal trials (~ 75 kg Yorkshire swine) at healthy baseline before and after pVAD placement and then following induction of cardiogenic shock. Animals were maintained according to NIH and AAALAC guidelines (CBSET, Lexington, MA). Body temperature, oxygen saturation, end-tidal carbon dioxide concentration, and three-lead electrocardiogram were continuously monitored. Animals underwent anesthesia induction via intramuscular injection of tiletamine–zolazepam (4–6 mg/kg) and then maintained on continuous sedation with intravenous propofol (~ 0.2–0.4 mg/kg/min). Ventilation was maintained in volume control mode using a Puritan Bennett 840 Ventilator (Medtronic, Dublin, Ireland) with a tidal volume of 8 ml/kg body weight and fraction of inspired oxygen of 50% and an initial positive end-expiratory pressure (PEEP) of 5 cmH_2_O. An arterial partial pressure of carbon dioxide of 35–45 mmHg was maintained by titrating respiratory rate. Prior to study interventions, animals were volume loaded until central venous pressure was > 8 mmHg to ensure tolerance to study procedures and clean data collection.

Hemodynamic measurements were obtained through femoral artery and vein, carotid artery, and jugular vein access points and were continuously recorded using a research-grade data acquisition system (ADInstruments, Dunedin, New Zealand). Pressure–volume conductance catheters and straight-tip pressure sensors (Millar, Houston, TX) obtained ventricular pressures and volumes and arterial pressures, respectively. Cardiac output was obtained via triplicate thermodilution measurements, and venous pressures via femoral vein external transducer (ADInstruments, Dunedin, New Zealand). Fluoroscopy was used to advance and verify pVAD placement.

### Mechanical circulatory support device

The Impella CP (Abiomed, Danvers, MA) was used as the paradigmatic pVAD. Consisting of a transvalvular, catheter-mounted, mixed-flow pump, the device is introduced percutaneously, typically via the femoral artery, and advanced retrograde across the aortic valve such that blood is continuously pumped antegrade for LV unloading and increased perfusion. For this study, the pVAD was maintained on performance-level P4, corresponding to a speed of 35,000 revolutions per minute, which was set by an external controller that acts to modulate power delivered to the pump.

### Animal model and ventilator stimulus

Heart–lung interactions were assessed in three states in response to variable positive end-expiratory pressure (PEEP), starting at 5 cmH_2_O and then with progressive increases by intervals of 5 cmH_2_O, as tolerated by systemic hemodynamics. In the setting of deteriorating hemodynamics, defined as rapid and progressive decrease in mean arterial pressure (MAP) to less than 30 mmHg, PEEP was then returned to 5 cmH_2_O to permit animal recovery. Responses to ramping PEEP were first assessed in the healthy, unassisted heart as a control, followed by the healthy heart with pVAD support. Lastly, the response was assessed in cardiogenic shock with continued pVAD support. Diffuse LV ischemia was induced via injections of 0.25 mL compressible microspheres (diameter 45–105 µm) (Hydropearl, Terumo, Tokyo, Japan) mixed with 10 mL each of isotonic saline and contrast into the left anterior descending coronary artery through a Judkins left catheter. Injections were continued to reach cardiogenic shock, defined by LV end-diastolic pressure (EDP) > 20 mmHg, MAP < 60 mmHg and/or mixed venous oxygen saturation < = 55%. If needed, access to the left circumflex was obtained for additional injections of microspheres, mindful of animal state and degree of left anterior descending occlusion.

### Data analysis

Data were registered and analyzed using MATLAB (Mathworks, Natick, MA). Metrics were averaged over 30 s during hemodynamic stability at each state. One instance in which hemodynamic stability was not achieved and two instances in which catheter signals contained artifact which precluded analysis were excluded. Ventricular preload was assessed via EDP, end-diastolic volume (EDV), and compliance (EDV/EDP). Mean arterial pressure, vascular resistance, and vascular compliance were used to study ventricular afterload. Ventricular function was assessed using stroke volume, pulse pressure, stroke work, and dP/dt_max_ and dP/dt_min_, measures of contractility and relaxation. Ventricular-ventricular interactions were assessed using the ratio between RV and LV EDP, dP/dt_max_ and dP/dt_min_.

### Statistical analysis

Differences between groups were determined using a two-sample *t*-test with *p* < 0.05 denoting statistical significance. Statistical analysis was conducted using MATLAB and the Statistics and Machine Learning Toolbox from MATLAB. Additional details on statistical analysis, sample size, and outcome measures have been provided using the ARRIVE 2.0 guidelines (Supplemental Table 1).

## Results

The cardiovascular response to PEEP was assessed in five animal trials and three states per animal consisting of: health, health with pVAD support, and cardiogenic shock with pVAD support. In the healthy state, two animals tolerated 4 PEEP intervals, up to 20 cmH_2_O, and three animals tolerated 3 PEEP intervals, up to 15 cmH_2_O prior to onset of hemodynamic instability. Following pVAD placement, all five animals tolerated 3 PEEP intervals, up to 15 cmH_2_O. In the shock state, two animals tolerated 3 PEEP intervals, up to 15 cmH_2_O, and three animals tolerated 2 PEEP intervals, up to 10 cmH_2_O.

### Healthy baseline response to PEEP

Intrathoracic pressure affects venous return, the transmural pressure for both the heart and pulmonary vasculature, and the pressure gradient from the venae cavae into the right heart and from the left heart into the proximal aorta. Consequently, changes in intrathoracic pressure have profound impact on RV and LV loading states and function. The current study not only evaluated concomitant cardiac and pulmonary support but also added to classic hemodynamic metrics to incorporate ventricular volume measurements, noted as a particular challenge in the past, allowing for assessment of pressure–volume loops and recreation of the classic Frank-Starling curves.

**Fig. 1:**
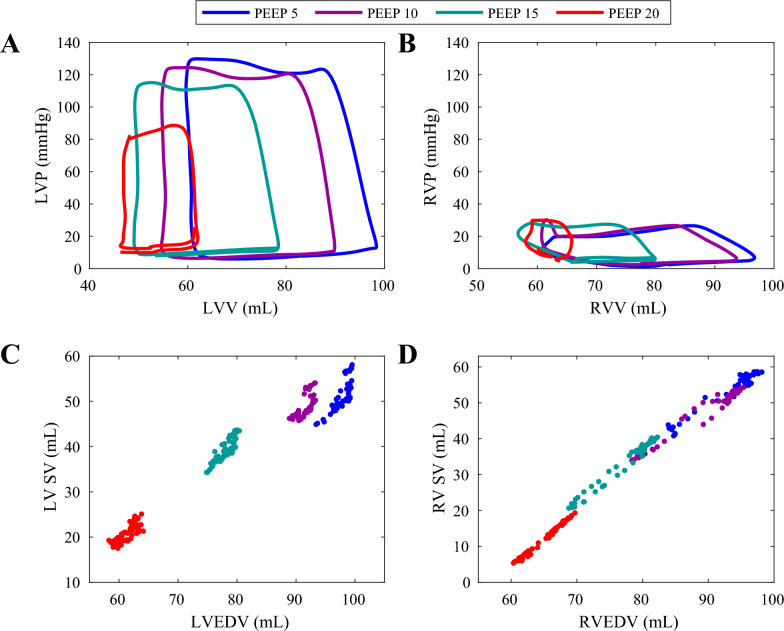
Impact of intrathoracic pressure on right and left ventricular physiologic state. **A** Left and **B** right ventricular pressure–volume loops across progressive PEEP ramps in health. Each loop is a representative, single cardiac cycle from one subject. Loops were extracted from the same timepoint in the respiratory cycle across each PEEP condition. Corresponding **C** left and **D** right ventricular Frank-Starling Curves between ventricular end-diastolic volume and stroke volume

Increasing PEEP profoundly reduced venous return to both ventricles (Fig. 1A, B). The corresponding effects on ventricular stroke volume yield a contiguous Frank-Starling relationship between stroke volume and end-diastolic volume (Fig. 1C, D). In the face of minimal effects on ventricular contractility, this must imply the existence of compensatory effects which allow for balance between ventricular elastance and arterial elastance with respect to ventricular preload. Pressure–volume loops revealed increased EDP for both ventricles despite reduced EDV, demonstrating the effect of increased PEEP on ventricular compliance. RV systolic pressures increased as a result of increased afterload in the pulmonary circulation while LV systolic pressures decreased greatly due to the combined effects of reduced filling, ventricular output, and afterload. Considering the holistic effects on ventricular function, both ventricles experienced progressive reductions in stroke work, with LV values of 2593, 2255, 1769, and 740 mmHg*mL across incremental PEEP conditions of 5, 10, 15, and 20 cmH_2_O, and RV values of 418, 436, 360, and 126 mmHg*mL, respectively. The slope of the stroke work reductions in the LV is 6.4 fold greater than that of the RV, demonstrating increased LV sensitivity to PEEP and preload-dependence compared to the RV (Supplemental Figure 1).

### Impact of MCS on heart–lung effects

**Fig. 2:**
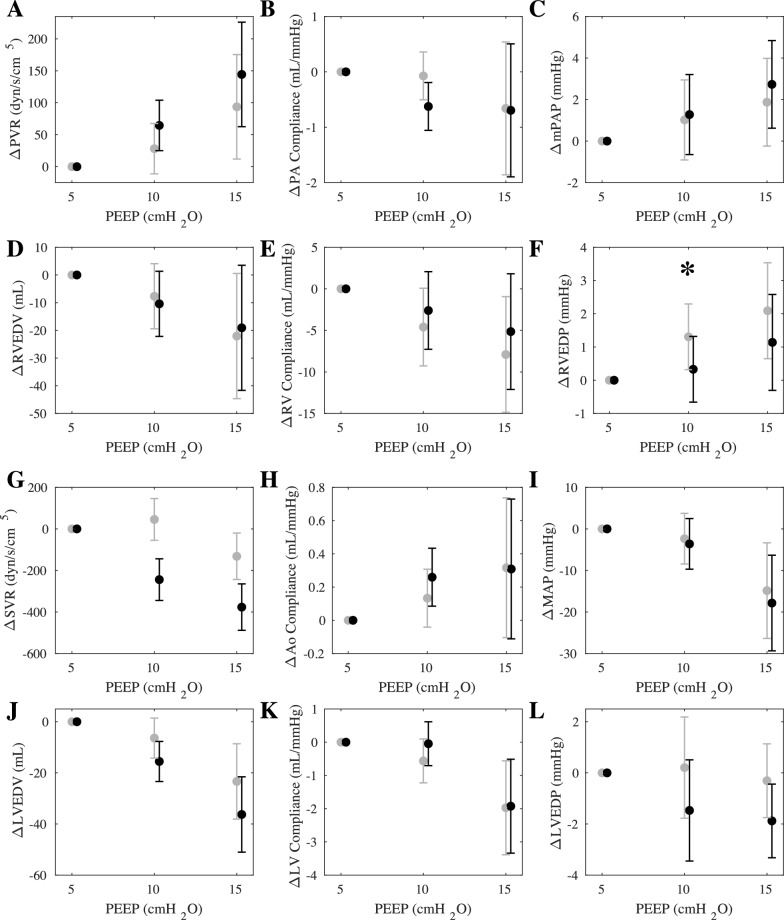
Cardiopulmonary impact on right and left heart loading states in health with and without pVAD support. Effects of progressive increases in PEEP with (black) and without (grey) pVAD support in health on cardiovascular function. Each condition was evaluated in all five animals, including analysis from cardiac cycles across 30 s of stable data in each animal. Metrics evaluated include the impact of PEEP on right ventricular afterload determinants, including **A** Pulmonary vascular resistance (PVR), **B** Pulmonary vascular compliance, and **C** Mean pulmonary artery pressure (mPAP), and preload determinants, including **D** RV end-diastolic volume (EDV), **E** RV Compliance, and **F** RV end-diastolic pressure (EDP). Corresponding effects on left ventricular afterload and preload determinants were similarly shown, including **G** Systemic vascular resistance (SVR), **H** Aortic compliance, **I** Mean arterial pressure (MAP), **J** LVEDV, **K** LV compliance, and **L** LVEDP. Values represent the average delta from baseline at PEEP 5, with error bars representing standard deviation across the animals. *p-value < 0.1

Left-sided pVAD support unloads the LV, reducing preload, increasing forward flow and organ perfusion, and promoting venous return to the heart. Introduction of the pVAD thereby alters the loading state of both ventricles. However, the effect of PEEP on cardiovascular state in health with and without pVAD support was highly consistent across the conditions (Fig. 2).

Pulmonary vascular resistance increased, and pulmonary vascular compliance decreased, leading to an increase in mean pulmonary artery pressure (mPAP) (Fig. 2A–C). Left ventricular afterload and output decreased, and in conjunction, MAP dropped (Fig. 2G–I). RV and LV EDV decreased with reduced venous return (Fig. 2D, J), and ventricular compliance decreased with reduced transmural pressure across the heart (Fig. 2E, K). Slightly more profound differential effects of PEEP on EDP were observed in the absence and presence of the pVAD. The pattern whereby PEEP increased RVEDP but with minimal change in LVEDP was reversed with the pVAD. In the presence of MCS RVEDP rise was muted and LVEDP fall was exaggerated, demonstrating perhaps greater unloading from the pVAD at higher PEEP and protection of RVEDP in face of reduced venous return (Fig. 2F, L).

### Changes in heart–lung response with variable cardiovascular loading states

**Fig. 3:**
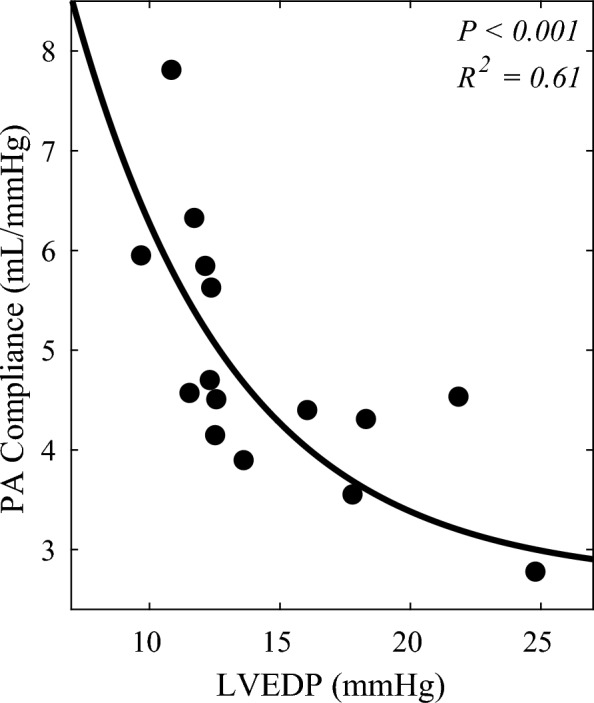
Link between left heart loading state and pulmonary vascular function. Correlation between pulmonary vascular compliance and left ventricular loading state, across the range of conditions modeled. *n* = 15 (5 animals, 3 conditions each, all at PEEP of 5 cmH_2_O)

Initiation of cardiogenic shock created different cardiovascular loading states and functions for the evaluation of heart–lung interactions. Generally, it was observed that LVEDP increased with LV ischemia and impairment. Due to backwards propagation of LV overload into the pulmonary circulation, pulmonary vascular compliance was reduced, with a continuum forming between LV load and pulmonary vascular function (Fig. 3). For example, on one end of the spectrum, there is a subject with LVEDP of 10.8 mmHg and pulmonary vascular compliance of 7.8 mL/mmHg, and on the other end of the spectrum, there is a subject with LVEDP of 24.8 mmHg and pulmonary vascular compliance of 2.8 mL/mmHg, demonstrating the negative relationship between these physiologic parameters.

**Fig. 4:**
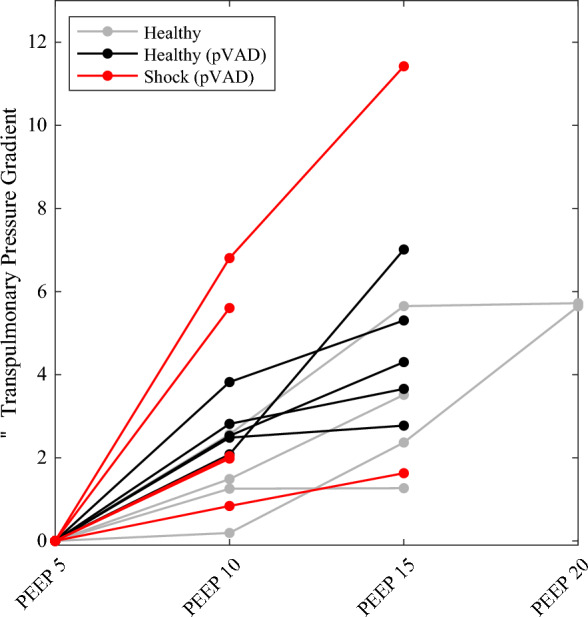
Transpulmonary response to PEEP. As PEEP was increased, the transpulmonary pressure gradient increased in response in all states. In the healthy state, two animals tolerated PEEP intervals up to 20 cmH_2_O, and three animals tolerated PEEP up to 15 cmH_2_O (*n* = 10). Following pVAD placement, all five animals tolerated PEEP up to 15 cmH_2_O (*n* = 10). In the shock state, two animals tolerated PEEP up to 15 cmH_2_O, and three animals tolerated PEEP up to 10 cmH_2_O (*n* = 7)

As we consider the influence of combined heart and lung support, one metric of particular interest is the transpulmonary pressure gradient, which serves to evaluate RV-LV serial interactions across the pulmonary vascular bed. This is determined by the difference between mPAP and LVEDP. Across all three conditions modeled, increased PEEP led to an increase in the transpulmonary pressure gradient (Fig. 4).

**Fig. 5:**
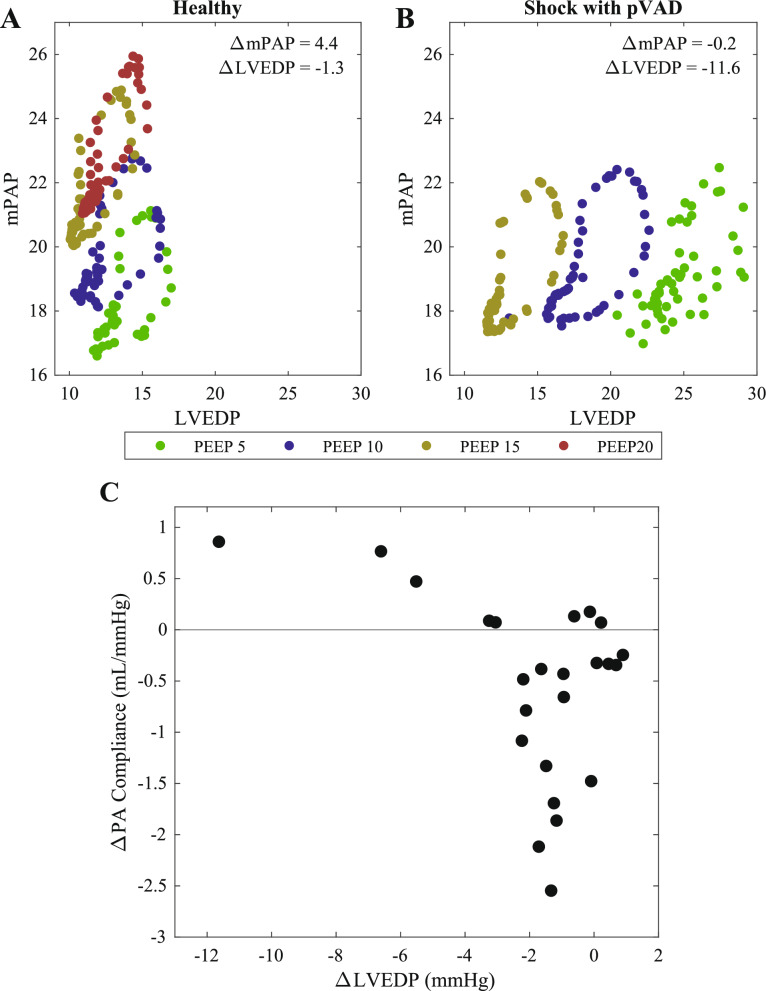
Link between left heart loading state and pulmonary vascular function. Correlation between pulmonary vascular compliance and left ventricular loading state, across the range of conditions modeled including **A** Health and **B** Cardiogenic shock.** C** Correlation between change in pulmonary vascular compliance as PEEP is increased with change in left ventricular loading state as PEEP is increased (*n* = 24)

Notably and moreover, the means of increasing the transpulmonary pressure gradient varied according to the loading conditions in the heart. To study this further, we drew upon previous work which explored hysteresis of the transpulmonary pressure gradient in conjunction with the respiratory cycle [31]. The increase in this pressure gradient was principally driven by an increase in mPAP of the healthy heart (Fig. 5A) and by a decrease in LVEDP at higher PEEPs in shock (Fig. 5B). These variable changes track with the observations in Fig. 2 and overall LVEDP dependence on loading state in concert with variable changes in pulmonary vascular compliance. With little change in LVEDP, the standard impact of PEEP occurred, meaning there was a decline in pulmonary vascular compliance (Fig. 5C). However, in the cases of significant decline in LVEDP, the effects of reduced volume load trumped the anticipated physiologic effects of PEEP, actually yielding an improvement in pulmonary vascular compliance (Fig. 5C).

### Functional implications of varied heart–lung response

**Fig. 6:**
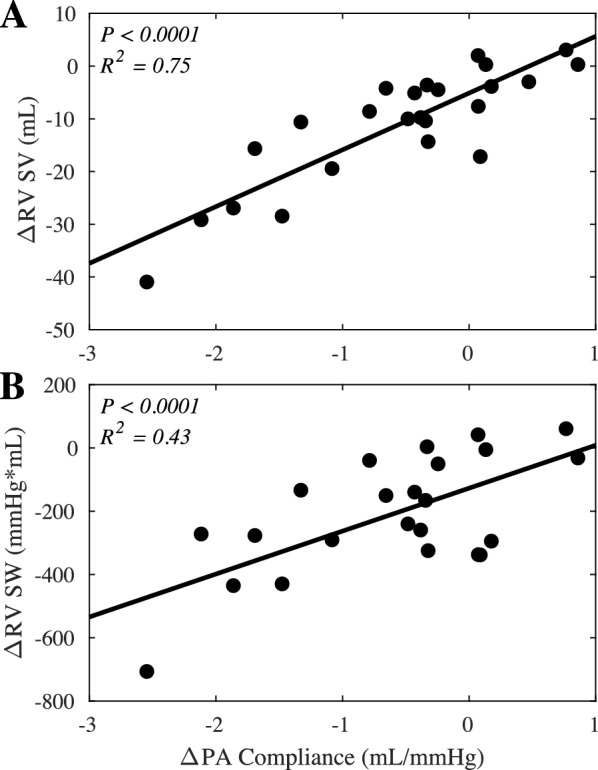
Variable right ventricular functional response to PEEP. The changes in **A** RV stroke volume (SV) (*n* = 24) and **B** RV stroke work (SW) correlate with the change in pulmonary vascular compliance as PEEP is increased (*n* = 22, 2 instances with catheter artifact were excluded)

Ultimately, the change in pulmonary vascular compliance also correlated with the right heart response to PEEP. With the standard case of reduced pulmonary vascular compliance, RV stroke volume and stroke work were reduced (Fig. 6). For example, in one subject with a pulmonary vascular compliance reduction of − 2.5 mL/mmHg, there was a corresponding reduction in stroke volume of 40.9 mL and in stroke work of 707 mmHg*mL. However, in the cases of increased pulmonary vascular compliance, this allowed a much milder change or even an increase in RV stroke volume and stroke work due to improved afterload (Fig. 6). For example, in a case where pulmonary vascular compliance increased by 0.77 mL/mmHg, there was a corresponding increase in stroke volume of 3.1 mL and in stroke work of 60.7 mmHg*mL.

## Discussion

The application of advanced engineering to the care of critically ill patients has spurred development of new mechanical devices capable of restoring systemic perfusion and homeostasis in the setting of profound organ injury [26, 27]. Although providing great benefit, these devices also impact native organ function and disrupt synchronized physiological mechanisms which complicates MCS use and demands deeper insight to maximize their efficacy. The lack of consistent observed benefit in clinical trials of MCS in cardiogenic patients adds urgency to the need for enhanced understanding of heart-device interactions and how to determine optimal management strategies to improve clinical outcomes [28–30].

One key challenge yet to be assessed is the effect of combined use of mechanical ventilators and pVADs to treat patients with concomitant heart and lung disease. Although changes in load and cardiovascular function due to intrathoracic pressure modulation have been previously examined [22–25], the addition of state-of-the-art technologies to further assess these impacts, initiation of MCS technologies to assess simultaneous device use, and implementation of disease models more representative of clinical scenarios allow for new understanding of heart–lung interactions and help support clinical management using these devices moving forward.

### Heart–lung interactions in health

Prior studies of the effects of intrathoracic pressure modulation on cardiac state through changes in PEEP are limited by lack of ventricular volume measurements [22–25]. To expand on previously reported findings, we measured biventricular pressure–volume relationships to determine stroke work and volume and enable generation of cardiac work and thermodynamic relationships at different basal intrathoracic pressures (Fig. 1). Increasing PEEP reduces ventricular preload while simultaneously increasing end-diastolic pressure (Fig. 2) consistent with reduced ventricular compliance likely due to the effect of increased intrathoracic pressure on restricting passive dilation of the ventricles during diastole. Simultaneously, RV and LV Frank-Starling curves remain constant across the examined PEEP interval range which suggests preserved ventricular-arterial elastance matching with a linear relationship between stroke volume and preload.

### Impact of MCS on heart–lung interactions in health and cardiogenic shock

Mechanical support from pVADs is increasingly used to sustain patients in circulatory failure. As pVAD and ventilator both alter RV and LV load, and simultaneous use is common, it is advantageous to understand how these ventricular-ventricular interactions may be impacted. Notably, in the healthy state, the traditional response observed by increasing PEEP remains similar with added pVAD support (Fig. 2). In health, this finding indicates that the native heart–lung response dominates over the impact of pVAD function. This is not the case in shock where effects are amplified and where MCS restores health in intuitive but perhaps unanticipated ways.

Myocardial infarction is the most common etiology of cardiogenic shock and serves to motivate our animal model in which left anterior descending artery microbead embolization induced severe reductions in LV contractility. In response to cardiogenic shock with a decreased in ventricular contractile reserve and reduced forward flow, LVEDP rose. As a result of backwards failure, a continuum formed between LV loading state and pulmonary vascular function (Fig. 3). Increasing load in the left ventricle also led to increased volume in the pulmonary circulation. This resulted in reduced pulmonary vascular compliance as the circulation reached its limit of distensibility.

These variable loading states then led to variable responses to increasing PEEP. In the healthy state, there was little change in LVEDP, and therefore, increased mPAP drove an increase in transpulmonary pressure gradient (Figs. 4, 5). In contrast, in the case of LV volume overload, reduced venous return from increasing PEEP resulted in a significant decline in LV loading state, therefore reducing volume overload in the LV and pulmonary circulation. As a result, mPAP actually declined. Mechanical ventilation with pVAD support restored the responses to a more health-like state.

Although the changes in all states manifested as an increase in transpulmonary pressure gradient, the corresponding driver of that change (mPAP vs. LVEDP) resulted in impactful consequences for loading states and function. In the traditional case, increased PEEP yields increased mPAP and a reduction in PA compliance (Fig. 5). In the case of volume overload though, increased PEEP actually improved LV loading state in the face of cardiogenic shock. This also improved loading state in the pulmonary vascular bed, ultimately enabling an increase in pulmonary vascular compliance.

### Clinical impact of varied heart–lung response

The use of mechanical support devices disrupts native cardiovascular interactions, enabling manipulation of cardiovascular loading states and blood flow in ways that were previously impossible. While this creates the risk of decompensation and worsened function, this also creates an incredible opportunity to optimize cardiovascular states. In particular, we note here cases of concomitant cardiac and pulmonary disease which might require combined circulatory and ventilatory support. Combined used in the healthy case presented little change compared to traditional understanding of heart–lung interactions, likely due to the dominance of native function. However, with disease and disrupted loading states, results show that mechanical ventilation could aid cardiac support, serving to both unload the LV and promote increased pulmonary vascular compliance. As explored in previous work, this may then improve right heart tolerance to LV unloading [31].

Since the effect of PEEP is dependent on the volume state, this effect could be particularly powerful in the case of intravascularly overfilled patients. Traditionally, PEEP increases central venous pressure (CVP) by raising intrathoracic pressure, which is transmitted to the thoracic veins and right atrium. When CVP is low (preload-dependent state), the increase in intrathoracic pressure from PEEP can significantly reduce venous return, leading to a marked decrease in cardiac output. In contrast, in overfilled patients, the reduction in venous return and cardiac output can be less pronounced, as the elevated filling pressures can partially offset the impedance to venous return caused by PEEP. This relationship is central to optimizing PEEP settings in critically ill patients, as excessive PEEP in hypovolemic states can precipitate hemodynamic compromise, but PEEP can become a powerful tool for volume management and LV unloading, in conjunction with pVAD support, in the case of volume overloaded patients. Using ramp studies of both the ventilator and pVAD, and tracking filling pressures could provide an approach for optimization of right–left ventricular states to maximize cardiac output.

### Limitations and future work

The results presented constitute a preliminary study in a preclinical porcine model of cardiac intervention. The experimental findings detail the complex interventricular interactions that are affected by the simultaneous presence of left-sided MCS and mechanical ventilation. This initial study sought to improve understanding of the effects of varying intrathoracic pressure via PEEP changes on hemodynamics and metrics of ventricular function. Notably, a distinct protocol was followed for the microembolization model used, which allows for rigor in the experimental process, but does not recapitulate the full range of physiology seen in cardiogenic shock patients nor the range of interventions that made be coupled with mechanical support in the clinic. Additionally, this set of preliminary experiments relied on animals with normal lung function which limits the direct clinical translation of the reported findings to patients on pVAD support intubated for airway protection. Future work will look to extend these findings in more complex model systems of combined cardiac and pulmonary injury, and further evaluation of these findings in the clinic will be required to ensure validation and extension of this work in patients.

## Supplementary Information

Additional file 1.

Additional file 2.

## Data Availability

Data will be made available upon reasonable request.

## Abbreviations

MCS: Mechanical circulatory support
pVAD: Percutaneous ventricular assist device
LV: Left ventricle
RV: Right ventricle
PEEP: Positive end-expiratory pressure
MAP: Mean arterial pressure
EDP: End-diastolic pressure
EDV: End-diastolic volume
mPAP: Mean pulmonary artery pressure
