# Supplementary material for: Combined mechanical ventilatory and mechanical circulatory support aids pulmonary vascular state in cardiogenic shock
Source: Intensive Care Med Exp. 2025 Oct 15;13:100. doi: 10.1186/s40635-025-00811-2 (PMC12528517; doi:10.1186/s40635-025-00811-2)
Supplement: Supplementary file 1 — Additional file 1. [file 40635_2025_811_MOESM1_ESM.docx]

**Supplemental Figure 1 Legend: Impact of PEEP on Ventricular Stroke Work** – As PEEP was increased, the stroke work in both ventricles decreased. The effect on LV stroke work (red) was 6.4 fold higher than the effect on RV stroke work (blue). Values represent the corresponding stroke work values from the pressure-volume loops in Figure 1.

**Supplemental Table 1 : ARRIVE 2.0 Checklist –** Essential 10 items to be included in the manuscript and section and line numbers.

| **Item** | **Recommendation** | **Section/line number, or reason for not reporting** |
| --- | --- | --- |
| **Study Design** | For each experiment, provide brief details of study design including:   1. The groups being compared, including control groups. If no control group has been used, the rationale should be stated. 2. The experimental unit (e.g. a single animal, litter, or cage of animals). | **Methods:** Animal Preparation and Data Acquisition &  **Methods:** Animal Model and Ventilator Stimulus |
| **Sample Size** | 1. Specify the exact number of experimental units allocated to each group, and the total number in each experiment. Also indicate the total number of animals used. 2. Explain how the sample size was decided. Provide details of any a priori sample size calculation, if done. | **Methods:** Animal Preparation and Data Acquisition & **Results** |
| **Inclusion and Exclusion Criteria** | 1. Describe any criteria used for including and excluding animals (or experimental units) during the experiment, and data points during the analysis. Specify if these criteria were established a priori. If no criteria were set, state this explicitly. 2. For each experimental group, report any animals, experimental units or data points not included in the analysis and explain why. If there were no exclusions, state so. 3. For each analysis, report the exact value of n in each experimental group. | **Methods:** Data Analysis, **Figure Legends** |
| **Randomization** | 1. State whether randomisation was used to allocate experimental units to control and treatment groups. If done, provide the method used to generate the randomisation sequence. 2. Describe the strategy used to minimise potential confounders such as the order of treatments and measurements, or animal/cage location. If confounders were not controlled, state this explicitly. | Not applicable |
| **Blinding** | Describe who was aware of the group allocation at the different stages of the experiment (during the allocation, the conduct of the experiment, the outcome assessment, and the data analysis). | Not applicable |
| **Outcome Measures** | 1. Clearly define all outcome measures assessed (e.g. cell death, molecular markers, or behavioural changes). 2. For hypothesis-testing studies, specify the primary outcome measure, i.e. the outcome measure that was used to determine the sample size. | **Methods:** Data Analysis |
| **Statistical Methods** | 1. Provide details of the statistical methods used for each analysis, including software used. 2. Describe any methods used to assess whether the data met the assumptions of the statistical approach, and what was done if the assumptions were not met. | **Methods:** Statistical Analysis |
| **Experimental Animals** | 1. Provide species-appropriate details of the animals used, including species, strain and substrain, sex, age or developmental stage, and, if relevant, weight. 2. Provide further relevant information on the provenance of animals, health/immune status, genetic modification status, genotype, and any previous procedures. | **Methods:** Animal Preparation and Data Acquisition |
| **Experimental Procedures** | For each experimental group, including controls, describe the procedures in enough detail to allow others to replicate them, including:   1. What was done, how it was done and what was used. 2. When and how often. 3. Where (including detail of any acclimatisation periods). 4. Why (provide rationale for procedures). | **Methods:** Animal Preparation and Data Acquisition & **Methods:** Animal Model and Ventilator Stimulus |
| **Results** | For each experiment conducted, including independent replications, report:   1. Summary/descriptive statistics for each experimental group, with a measure of variability where applicable (e.g. mean and SD, or median and range). 2. If applicable, the effect size with a confidence interval. | **Figures 2, 3, and 6** |
