# Supplementary figures and images for: Combined mechanical ventilatory and mechanical circulatory support aids pulmonary vascular state in cardiogenic shock
Source: Intensive Care Med Exp. 2025 Oct 15;13:100. doi: 10.1186/s40635-025-00811-2 (PMC12528517; doi:10.1186/s40635-025-00811-2)

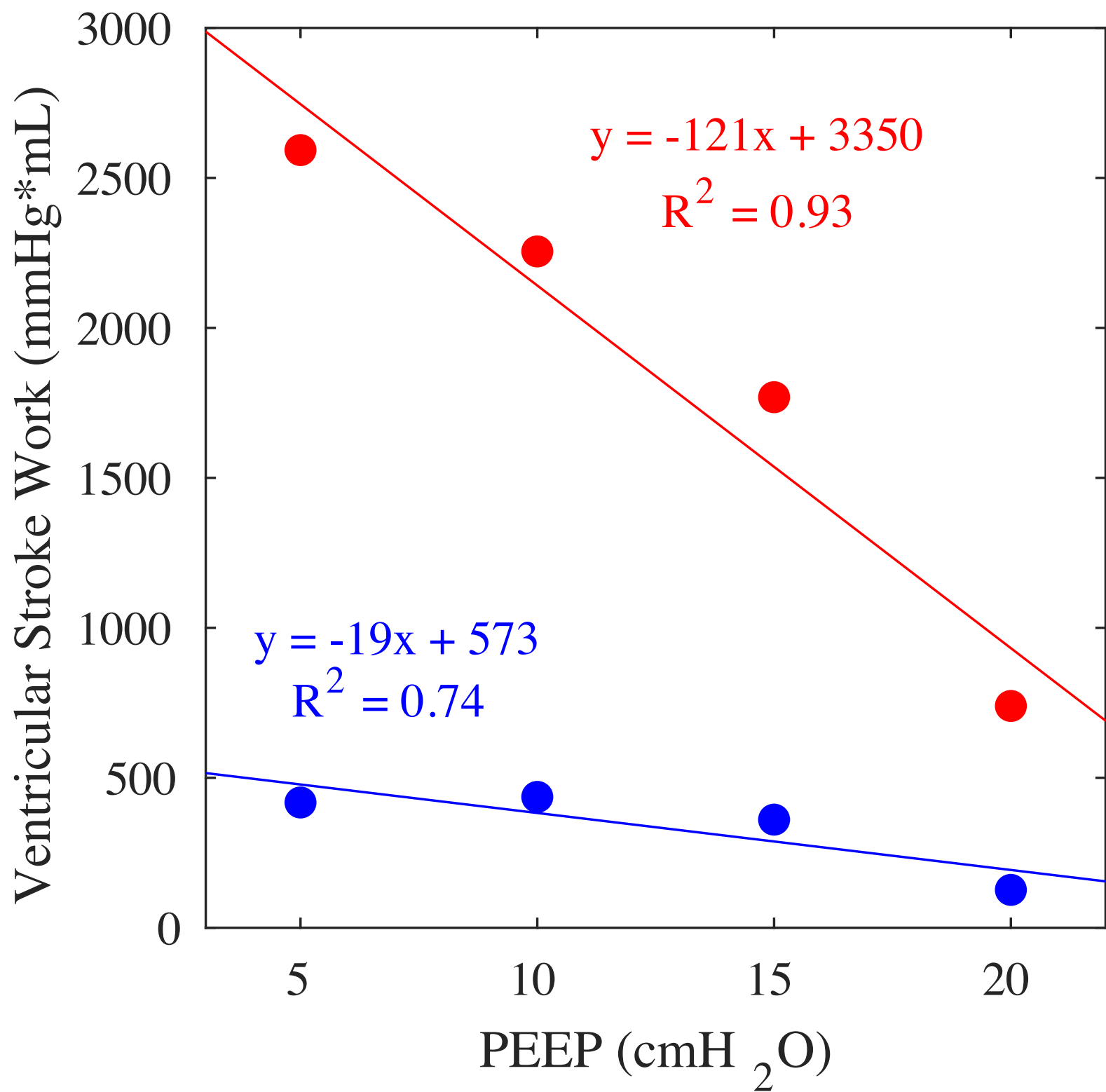

Supplement: Supplementary file 2 — Additional file 2. [file 40635_2025_811_MOESM2_ESM.pdf]
